# Elevated Cytokine Levels in Aqueous Humor Are Associated with Peripheral Anterior Synechiae after Penetrating Keratoplasty

**DOI:** 10.3390/ijms222212268

**Published:** 2021-11-12

**Authors:** Yuki Kusano, Takefumi Yamaguchi, Sota Nishisako, Takehiro Matsumura, Masaki Fukui, Kazunari Higa, Toshihiro Inoue, Jun Shimazaki

**Affiliations:** 1 Department of Ophthalmology, Ichikawa General Hospital, Tokyo Dental College, Chiba 2728-513, Japan; 081m1037@gmail.com (Y.K.); nishisako@eyebank.or.jp (S.N.); takebou_mail@yahoo.co.jp (T.M.); fu_kky@hotmail.com (M.F.); higa@eyebank.or.jp (K.H.); jun@eyebank.or.jp (J.S.); 2 Department of Ophthalmology, Kumamoto University, Kumamoto 8608-556, Japan; noel@da2.so-net.ne.jp; 3 Cornea Center Eye Bank, Ichikawa General Hospital, Tokyo Dental College, Chiba 2728-513, Japan

**Keywords:** aqueous humor, corneal transplantation, cytokine, glaucoma, intraocular pressure, penetrating keratoplasty, peripheral anterior synechia, iris atrophy

## Abstract

Peripheral anterior synechiae (PAS) after corneal transplantation leads to refractory glaucoma and permanent loss of vision. However, the exact mechanism remains elusive. This study aimed to evaluate the association between cytokine levels in the aqueous humor (AqH) and the progression of PAS after penetrating keratoplasty (PKP). We measured 20 cytokine levels in AqH and assessed the correlation with PAS progression after PKP in 85 consecutive patients who underwent PKP. We also evaluated age-dependent alterations in PAS and cytokine levels in DBA2J mice. PAS developed in 38 (44.7%) of 85 eyes after PKP. The incidence of intraocular pressure increase after PKP was significantly greater in eyes with PAS (26.3%) than in those without PAS (2%, *p* = 0.0009). The PAS area at 12 months after PKP was significantly positively correlated with the preoperative levels of interleukin (IL)-6, interferon (IFN)-γ and monocyte chemotactic protein (MCP)-1 (*p* ≤ 0.049). In the DBA2J mice, an experimental glaucoma model that developed PAS at 50 weeks, the AqH levels of IL-2, IL-6, IL-10, IFN-γ, tumor necrosis factor-α, MCP-1 and granulocyte-macrophage colony-stimulating factor (GM-CSF) significantly increased at 50 weeks compared to 8 weeks (*p* ≤ 0.021). In conclusion, inflammatory alterations in the AqH microenvironment, such as high preoperative specific cytokine levels, can lead to PAS formation and glaucoma.

## 1. Introduction

Glaucoma is a critical complication after corneal transplantation that can lead to permanent loss of vision. The incidence of glaucoma varies among surgical procedures; 9–35% after penetrating keratoplasty (PKP) [1,2,3,4], 0–4.5% after anterior lamellar keratoplasty (ALK) [5,6,7] and 2–14% after endothelial keratoplasty (EK) [8,9,10,11,12]. The trends of postoperative transient intraocular pressure (IOP) increase also vary among these procedures, that is, 29–80% after PKP, 17–36% after ALK and 31–60% after EK [1,2,3,4,5,6,7,8,9,10,11,12]. Various mechanisms are involved in elevated IOP, including intraoperative viscoelastic material, pupillary block due to air tamponade, response to topical steroids, damage to outflow mechanisms and angle-closure due to peripheral anterior synechiae (PAS) [1,9]. PAS is known to cause refractory glaucoma after corneal transplantation [13,14,15], iridocorneal endothelial (ICE) syndrome [16,17] and uveitis [18]. However, the exact mechanism of PAS development remains poorly understood.

Recent advances in anterior segment optical coherence tomography (AS-OCT) have enabled the non-invasive and accurate quantification of PAS [13,14,15,19,20,21,22,23]. Using state-of-the-art AS-OCT, we have demonstrated that the elevated levels of specific cytokines in the aqueous humor (AqH) were significantly correlated with the development of PAS after EK [24]. This suggests that the inflammatory microenvironment in AqH leads to PAS development and refractory glaucoma after corneal transplantation. However, the association between AqH cytokine levels and PAS after PKP has not yet been documented. Thus, we hypothesized that high AqH total protein and cytokine levels may be associated with PAS formation after PKP. In the current study, we measured AqH protein and cytokine levels before PKP and quantified PAS after PKP using AS-OCT. Second, we evaluated the correlations between AqH total protein/cytokine levels and PAS severity. Third, we evaluated PAS formation in a mouse model with an age-dependent elevation of cytokines in the AqH.

## 2. Results

### 2.1. PAS after PKP

**Figure 1:**
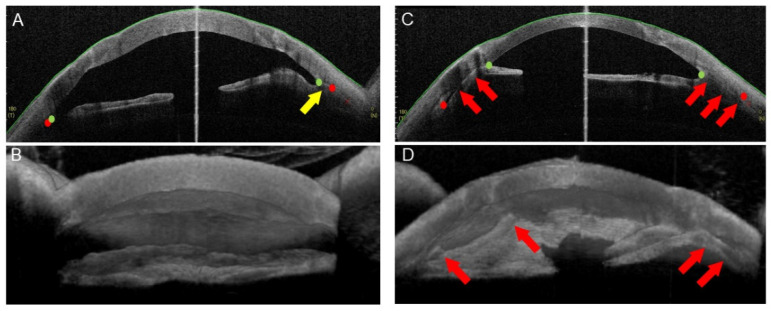
Three-dimensional anterior segment OCT analysis of ITC before and after PKP. Anterior segment OCT images of a representative patient before and after PKP. A 64-year-old woman with bullous keratopathy successfully underwent PKP. The total protein level in the aqueous humor was 1.55 mg/mL (0.3–0.4 mg/mL in normal eyes). Before PKP (**A**,**B**), the patient had limited ITC on the nasal side (yellow arrows). After PKP (**C**,**D**), ITC developed on the nasal side and expanded on the temporal side (red arrows). The red points represent the scleral spurs. The green points indicate the peripheral endpoint of the iris.

Our recent study demonstrated a positive correlation between PAS formation and cytokine levels after EK [24]. In the current study, we first sought to quantify PAS after PKP in our hospital. A total of 87 consecutive patients who underwent PKP at Tokyo Dental College Ichikawa General Hospital between October 2015 and January 2019 were included in this study (Table S1). All PKP surgeries were successful, without any serious intraoperative complications. After PKP, PAS developed in eight eyes (9.2%) without preoperative PAS and progressed in 16 eyes (18.4%) with preoperative PAS (Figure 1). PAS did not progress in 12 eyes (13.8%) with preoperative PAS. To evaluate PAS severity, we measured the degree, height and area of irido-trabecular contact (ITC) using three-dimensional AS-OCT, as previously reported [24]. The ITC indices (ITC %degree, maximal height and area) gradually increased after PKP; however, all ITC indices significantly increased at 12 months after PKP, compared to the preoperative values (Table 1, *p* < 0.03). 

### 2.2. Influence of PAS on Intraocular Pressure after PKP

PAS formation causes elevated IOP, leading to glaucoma. To confirm the influence of PAS on IOP after PKP, we compared the incidence of postoperative IOP rise, stratifying the participants based on the presence of PAS. Among these 87 eyes (Table 2), the incidence of IOP increased, higher than 21 mmHg was significantly greater in eyes with PAS (10 eyes (26.3%) out of 38) than in those without PAS (one eye (2%) out of 49 eyes, *p* = 0.0009). Next, to identify the clinical factors associated with PAS progression, we conducted multivariate analysis (Table S2). We selected the progression of the ITC area as it represented a three-dimensional alteration of ITC and was the most reliable PAS index [24]. It showed that only the presence of preoperative ITC was significantly associated with the progression of ITC area (β = 2.30, *p* < 0.0001), whereas preoperative total protein levels, axial length, graft size and patient age were not.

### 2.3. Association between PAS and Preoperative Cytokine Levels in the AqH

Inflammation in the AqH plays a pivotal role in PAS formation, leading to an IOP increase after corneal transplantation [24]. Since specific cytokine levels were found to be significantly correlated with PAS formation after EK [24], we compared the inflammatory cytokine levels in the AqH between the eyes with and without PAS after PKP (Table 3), which demonstrated that the levels of interleukin (IL)-6, IL-8, IL-10, IL-12p70, monocyte chemotactic protein (MCP)-1, P-selectin and interferon-gamma inducible protein (IP)-10 were significantly higher in eyes with postoperative PAS than in those without postoperative PAS (*p* ≤ 0.041). We further evaluated the correlation between cytokine levels in the AqH and ITC areas (Table 4). The results showed that the preoperative ITC area was significantly positively correlated with the preoperative levels of IL-6, IL-8 and IL-10 (*p* ≤ 0.026). Furthermore, the ITC area at 3 months was significantly positively correlated with the preoperative AqH levels of IL-6, IL-8 and IL-12p70 (*p* ≤ 0.014). The ITC area at 6 months was significantly correlated with the preoperative AqH levels of IL-4, IL-6, IL-10, IL-12p70, MCP-1, interferon (IFN)-γ, P-selectin and soluble intercellular adhesion molecule (sICAM)-1 (*p* ≤ 0.049). The ITC area at 12 months was significantly correlated with the preoperative AqH levels of IL-4, IL-6, MCP-1, IFN-γ and sICAM-1 (*p* ≤ 0.046). 

### 2.4. PAS and Preoperative Cytokine Levels in the AqH in a Mouse Model

**Figure 2:**
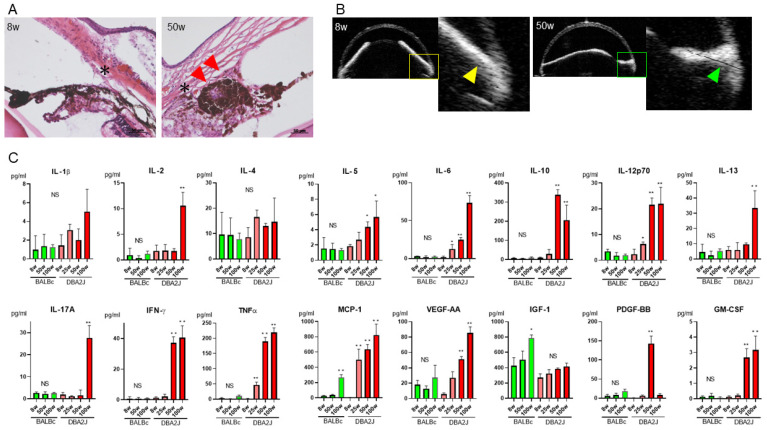
Age-dependent increase of cytokine levels in AqH in DBA2J mice. (**A**) The angle structure and iris tissue were normal in DBA2J at 8 weeks (* Schlemm’s canal); however, peripheral synechiae (PAS), iris nodules and iris atrophy developed at 50 weeks (red arrows). Scale bars: 50 μm. (**B**) In vivo anterior segment optical coherence tomography showed the absence of PAS at 8 weeks (white arrowheads), whereas PAS developed at the age of 50 weeks (green arrowhead). (**C**) The AqH levels of IL-2, IL-5, IL-6, IL-10, IL-12p70, IL-13, IL-17A, IFN-γ, TNF-α, MCP-1, PDGF-BB and GM-CSF were significantly elevated at 50 and 100 weeks in DBA2J, compared to 8 weeks in DBA2J. * *p* < 0.05, ** *p* < 0.001.

Previous reports have shown that the DBA2J mouse strain develops anterior segment anomalies with pigment dispersion, raised IOP, iris atrophy and glaucomatous damage [25]. We recently showed an age-dependent elevation of total protein levels in the AqH of DBA2J [23]. However, the association between AqH and PAS in DBA2J has not yet been investigated. Histopathological evaluation revealed that PAS, iris nodules and iris atrophy developed at the age of 50 weeks of DBA2J (Figure 2A), whereas normal Schlemm’s canal and trabecular meshwork were observed at 8 weeks. In vivo AS-OCT of DBA-2J also showed a decentered pupil and PAS at the age of 50 weeks, whereas there was no PAS in young DBA2J (Figure 2B). Next, we measured AqH levels in 16 cytokines (IL-1β, IL-4, IL-6, IL-8, IL-10, IL-12p70, IL-13, IL-17A, MCP-1, tumor necrosis factor (TNF)-α, IFN-γ, sICAM-1, granulocyte-macrophage colony-stimulating factor (GM-CSF), vascular endothelial growth factor (VEGF)-AA, insulin-like growth factor (IGF)-1, platelet-derived growth factor (PDGF)-BB) of DBA2J and BALB/c. The AqH levels of IL-2, IL-6, IL-10, IL-12p70, IL-13, IL-17A, IFN-γ, TNF-α, MCP-1, VEGF-AA, PDGF-BB and GM-CSF were significantly elevated at 50 and 100 weeks in DBA2J, compared to 8 weeks (Figure 2C). However, there was no significant IOP elevation between 8 (9.8 ± 0.8 mmHg) and 50 weeks (10.4 ± 0.5 mmHg, *p* = 0.21). Collectively, as post-PKP PAS formation was found in humans, DBA2J develops PAS, concomitant with specific cytokine elevation in AqH in an age-dependent manner.

## 3. Discussion

Post-keratoplasty glaucoma is a serious complication. The visual outcome significantly worsens in patients with glaucoma after corneal transplantation owing to irreversible loss of the visual field and increased risk of graft failure [26]. PAS was significantly associated with glaucoma development after both PKP and EK [27]. PAS is associated with chronic inflammation in the anterior chamber and breakdown of the blood-aqueous barrier (BAB) [28,29,30]. We recently reported that PAS formation after EK was significantly associated with high preoperative levels of IL-6, IL-8, MCP-1, IFN-γ and sICAM-1 in the AqH [24]. The current study on PKP showed similar trends in EK: First, in human participants, PAS formation after PKP was associated with high preoperative AqH levels of IL-6, MCP-1 and IFN-γ, and secondly, in an animal model that develops iris atrophy with age, PAS develops with the elevation of AqH IL-2, IL-6, IL-10, IFN-γ, TNF-α, MCP-1 and GM-CSF. Although cytokine elevation in AqH may not be the direct cause of PAS formation, we believe that the animal model will be useful for investigating the spatiotemporal mechanism.

MCP-1 is the main chemotactic factor for the macrophage migration and pathogenesis of chronic inflammation [21]. In patients with open-angle glaucoma, Inoue et al. showed that a higher preoperative MCP-1 level was associated with poorer outcomes of trabeculectomy in eyes with open-angle glaucoma [31]. Furthermore, Ohira et al. reported that MCP-1 levels were higher in uveitic glaucoma than in open-angle glaucoma [32]. Although they did not evaluate ITC in their study, we postulated that the MCP-1 level can directly result in PAS formation, followed by IOP elevation after trabeculectomy or uveitic glaucoma. However, as shown in previous studies in humans and animals, MCP-1 levels in AqH are elevated after cataract surgery, as proliferated lens epithelial cells on the capsule secrete MCP-1 over time after phacoemulsification [33]. 

IFN-γ is a pro-inflammatory cytokine that strengthens Th-1 immune response [34]. Previous studies have suggested that serum IFN-γ level can be a risk factor for various systemic diseases, such as angina pectoris and sepsis, predicting major coronary events and mortality [35,36]. In the field of ophthalmology, numerous reports have evaluated IFN-γ levels in the AqH in various eye diseases and found significant elevations in uveitis, age-related macular degeneration (AMD), bullous keratopathy and eyes with graft immunological rejection [23,37,38,39]. Maier et al. showed that the IFN-γ level in AqH before PKP was higher in eyes with postoperative immune reactions than in those without immune reactions [39]. We previously revealed that IFN-γ levels in the AqH were elevated in eyes with bullous keratopathy [23]. However, this is the first report to demonstrate an association between PAS formation and IFN-γ levels in AqH. However, eyes with AMD and BK usually do not develop PAS; thus, the mechanism of PAS formation needs to be investigated in the future by identifying the source of increased IFN-γ levels and evaluating the influence of disrupted BAB of the iris stroma. IL-17A+ Th17 cells produce IFN-γ and mediate ocular surface autoimmunity. 

In the current study, IL-6 and MCP-1 levels were associated with both the presence of PAS and PAS area. In contrast, the levels of IL-10, IL-12p70, IP-10 and P-selectin were associated with the presence of PAS, but not with the PAS area at any time point. Owing to the complicated correlations among cytokine levels (Table S3), careful attention should be paid not to overinterpret the results; the elevated total protein and cytokine levels measured in the current study may not be the direct cause of PAS formation. The results of the current study suggest that a specific molecular mechanism is involved in PAS formation after corneal transplantation, leading to refractory glaucoma. We believe that we can avoid refractory glaucoma due to PAS formation after corneal transplantation in the future in two ways. First, minimizing iris damage during intraocular surgery will potentially prevent PAS formation, as the severity of iris damage is strongly positively correlated with chronically elevated levels of inflammatory cytokines in the AqH [29]. Second, we need to identify therapeutic molecules that can prevent PAS formation in the future. We speculate that a multi-omics approach is a powerful tool that can detect alterations in more than 200–1000 proteins and 100 metabolites in the AqH and 40,000 mRNA changes in trabecular meshwork [23,40]. The DBA2J strain, an AqH-associated PAS formation model, enables spatio-temporal analysis of AqH proteomic analysis and transcriptomic analysis of the iris tissue to identify the time-course mechanism of PAS formation. Interestingly, unlike MCP-1, IL-6 and IFN-γ, the levels of IL-1β in AqH were significantly elevated in human eyes with PAS progression and DBA2J at 50 weeks; however, this was not correlated with the PAS area. This suggests that IL-1β levels were elevated non-specifically without any association with PAS development.

Donor-related factors could affect the results of this study. Aqueous humor and cornea undergo postmortem changes can affect the tissue. Napoli et al. evaluated postmortem central corneal thickness (CCT) using optical coherence tomography and showed that CCT changes in different ways based on open or closed eye modes [41]. Locci E et al. reported time-dependent changes in the metabolic profile of AqH in sheep [42]. In human corneal transplantation, Lass JH et al. evaluated donor-related risk factors, such as cause of death, death to refrigeration time, refrigeration to preservation time and death to preservation time, in order to determine the factors associated with corneal endothelial cell loss after DSAEK [43]. They found that a donor history of diabetes was associated with lower corneal endothelial cell density. We will conduct a comprehensive study to assess the donor-related factors that could be associated with PAS development after PKP in the near future.

Human leukocyte antigen (HLA) matching is recommended for other organ transplantations to provide the best opportunity for graft success. However, histocompatibility matching does not form part of the current corneal transplant allocation policy in the US, European or Asian countries. This is because the evidence supporting HLA typing for corneal transplantation remains unclear, with no international consensus.

This study had some limitations. First, this study included heterogeneous etiologies for surgical indications, which can cause bias. Second, IOP did not increase in the DBA2J strain, although it showed PAS formation. This was attributable to the fact that PAS formation was local and did not involve the entire angle. DBA2J has been reported to be an experimental glaucoma model because the DBA2J mouse strain develops pigment dispersion, raised IOP, iris atrophy, and glaucomatous damage [25]. Recently, Turner et al. evaluated as many as 118 DBA2J mice from 12 to 48 weeks of age and reported the problems of using them as an experimental glaucoma model: First, the IOP of DBA2J is unreliable because IOPs did not increase in all DBA2J [44]. Second, neurodegenerative changes in DBA2J are independent of IOP [44]. Thus, we consider that the DBA2J strain is a PAS formation model secondary to pigment dispersion, iris atrophy and elevated total protein and cytokine levels in the AqH. Finally, we used different panels of cytokines to investigate the development of PAS in humans and mice. This was partly due to the small amount of AqH in mice (2–3 μL per mice), in which cytokine measurement was impossible using Luminex. The Lunaris™, which was used for cytokine measurement in mice in the current study, required only 5 μL per sample. Thus, we used different panels in humans and mice. 

In conclusion, PAS progressed in 38 eyes (44.7%) after PKP, in which the incidence of glaucoma was significantly higher than in those without PAS formation. Furthermore, PAS severity (ITC area) at 12 months after PKP was significantly correlated with the preoperative AqH levels of IL-4, IL-6, MCP-1, IFN-γ and sICAM-1. Additionally, a glaucoma animal model, DBA2J, developed PAS and iris atrophy with age, and the AqH levels of IL-1β, IL-6, IL-10, IFN-γ, TNF-α, MCP-1 and GM-CSF at 50 weeks were significantly higher than those at 8 weeks. These results suggest that microenvironmental changes in AqH cause progression of PAS after PKP as a result of chronic inflammation with elevated levels of specific cytokines. 

## 4. Materials and Methods

### 4.1. Participants and Surgical Technique

This prospective study adhered well to the tenets of the Declaration of Helsinki. This study was approved by the institutional ethics review board of Tokyo Dental College Ichikawa General Hospital (I-15-42R). Written informed consent was obtained from all the participants before the intervention. A total of 85 eyes from 85 patients were included in the current study. The etiologies of PKP in the studied eyes included bullous keratopathy (BK, 32 eyes), scar (19 eyes), keratoconus (14 eyes), infection (seven eyes), corneal dystrophy (four eyes) and other causes (11 eyes). Sample size calculations were based on our previous studies, which estimated that 30–40% of patients undergoing PKP develop PAS postoperatively [15] and that cytokine correlation analyses require at least 70 patients [22]. Based on these assumptions, we included 80–90 consecutive patients in the current study. No participants with active inflammation, such as unresolved infection, were included in the current study.

PKP was performed according to our standard technique, as previously described [45]. Briefly, PKP was performed under retrobulbar anesthesia. The donor button was cut using a Barron punch trephine. A Hessburg–Barron suction trephine was used to cut a partial-depth, circular incision in the cornea, centered at the geometric center of the cornea. Excision of the recipient corneal button was completed using curved corneal scissors. The graft was sutured in place with a single-running 10–0 nylon suture with 24 bites in all eyes. Donor corneas were obtained from domestic or American eye banks. Histocompatibility matching was not performed. The typical trephination size was 7.5 mm for recipient eyes and 7.75 mm for donor grafts. At the end of the surgery, 2 mg of subconjunctival betamethasone was administered. In patients with significant lens opacity (16 eyes), standard extracapsular cataract extraction (15 eyes) and phacoemulsification and aspiration (one eye) were performed with implantation of an intraocular lens (IOL), followed by simultaneous PKP. After PKP, the patients were prescribed topical eye drops of levofloxacin (Cravit, Santen, Osaka, Japan) and betamethasone 0.1% (Sanbetazon, Santen) five times a day. The betamethasone eye drop was administered three times per day for up to 6 months after PKP in all eyes. All PKP procedures were successful and uneventful. After PKP, the logarithm of minimal angle resolution significantly improved from 1.50 ± 0.54 preoperatively to 0.62 ± 0.45 at 3 months, 0.52 ± 0.49 at 6 months and 0.46 ± 0.52 at 12 months (all, *p* < 0.0001). The corneal endothelial cell density (cells/mm^2^) of the graft decreased from 2655 ± 314 to 1971 ± 585 at 3 months, 1820 ± 675 at 6 months and 1498 ± 736 at 12 months (all, *p* < 0.0001).

### 4.2. AS-OCT Imaging

All patients underwent AS-OCT examination preoperatively and at 3, 6 and 12 months postoperatively. AS-OCT (SS-1000, CASIA, TOMEY, Nagoya, Japan) is a type of Fourier-domain OCT that uses a 1320 nm wavelength scanning laser source and a photodetector to detect wavelength-resolved interference signals. All eyes were imaged by trained technicians using an “angle analysis” protocol, which was composed of 128 radial B-scans, each with 512 A-scans (16-mm scan length). This allowed 360° imaging of the whole anterior segment in 2.4 s. Eyes receiving topical medications that affected pupil size were not included.

### 4.3. Measurement of the ITC Area, Degree, and Maximum Height

To examine the ITC, the angle structure was analyzed using AS-OCT as previously reported [24]. Briefly, the extent of ITC in each meridian was measured using built-in software after manual detection of the scleral spur and anterior irido-angle adhesion. The software automatically aligned the scleral spur and the iris endpoint of individual cross-sectional OCT images and then computed the ITC area (mm^2^), which was defined as the area bound below the iris endpoint and above the scleral spur. The parameters were automatically calculated: ITC %degree (%), that is, the percentage of degrees that presented with ITC of the total angle degrees analyzed, maximum ITC height (mm), that is, the maximum distance from the scleral spur to the iris endpoint, and ITC area (mm^2^). In the present study, angle analysis could not be performed in the upper or lower areas as the eyelids obstructed angle imaging in some patients. Therefore, the ITC was measured at a total range of 90° on the nasal and temporal sides of the horizontal line.

### 4.4. Animal Experiments

This study adhered to the Association for Research in Vision and Ophthalmology Statement for the Use of Animals in Ophthalmic and Vision Research. Mice that were 8- to 50 weeks old were purchased from Charles River Laboratories (Yokohama, Japan). We used 8- to 50-week-old BALB/c mice and DBA2J mice. Each animal was deeply anesthetized using an intraperitoneal injection of medetomidine (0.15 mg/kg), midazolam (2 mg/kg), and butorphanol (2.5 mg/kg) before surgical procedures (AqH sample collection) and AS-OCT measurement. IOP in mice was measured using TonoLab (Icare, Finland Oy, Vantaa, Finland) under general anesthesia.

### 4.5. Aqueous Humor Samples

Human AqH samples containing 100–300 μL were obtained under sterile conditions at the beginning of surgery after topical anesthesia in PKP surgery. First, paracentesis was performed on the clear cornea. AqH samples were obtained using a 27-gauge needle, taking care not to touch the iris, lens or corneal endothelium. The samples were centrifuged at 3000× *g* for 5 min. The supernatants were stored in silicon-coated Eppendorf tubes. In the mouse experiments, approximately 15 μL of AqH samples were collected using a 27-gauge needle through an oblique incision from four to six mice. AqH samples from mouse eyes were centrifuged at 3000 rpm for 5 min, and the supernatants were diluted with distilled water up to 30 μL. The AqH samples were collected and stored at −80 °C until measurements.

### 4.6. Total Protein and Cytokine Level Measurements

The total protein levels of the AqH samples were determined using a DC protein assay (Bio-Rad, Hercules, CA, USA) [28]. For cytokine measurements in human samples, the levels of IL-1α, IL-1β, IL-4, IL-6, IL-8, IL-10, IL-12p70, IL-13, IL-17A, MIP-1α, MIP-1β, MCP-1, TNF-α, IFN-α, IFN-γ, E-selectin, P-selectin, sICAM-1, IP-10 and GM-CSF in AqH samples were measured using a Luminex (ProcaPlex kit, Luminex, San Antonio, TX, USA) beads-based multiplex immunoassay according to a previous report without dilution [29]. For cytokine measurements in mice, AqH samples were triplicated and the levels of IL-1β, IL-2, IL-4, IL-5, IL-6, IL-10, IL-12p70, IL-13, IL-17A, IFN-γ, TNF-α, IFN-α, MCP-1, VEGF-Aa, IGF-1, PDGF-BB and GM-CSF were measured using Lunaris™ (Mouse Ophthalmology 4-plex cytokine, Mouse 12-plex cytokine, AYOXXA Biosystem GmbH, Cologne, Germany).

### 4.7. Statistical Analysis

The data were analyzed using STATA/IC 14.0, for iOS (StataCorp LP, College Station, TX, USA). The Shapiro–Wilk test was used to assess whether the data were normally distributed. Spearman’s correlation analyses were used to evaluate the correlations between the total protein and cytokine levels in the AqH and ITC %degree, maximum height and area. Fisher’s exact test was used to assess the differences in the incidence of post-PKP IOP rise (≥21 mmHg) between the groups with and without ITC. For multivariate analyses of the clinical factors that were correlated with the progression of the ITC area, we selected five independent clinical factors (preoperative total protein levels in AqH, preoperative ITC, axial length, graft size and age; variance inflation factors = 1.11–1.12) and conducted multiple linear regression analyses. The data are expressed as mean ± standard deviation. Statistical significance was set at *p* < 0.05.

## Figures and Tables

**Table 1:** Time course alteration in ITC indices.

ITC	Preop	3 Months	6 Months	12 Months
% Degree *p*-value *	22.6 ± 34.2	19.3 ± 31.7 0.14	25.3 ± 37.0 0.14	29.8 ± 39.3 0.03
Maximal height (mm) *p*-value *	0.94 ± 1.56	0.76 ± 1.23 0.64	0.90 ± 1.41 0.86	1.22 ± 1.65 0.01
Area (mm^2^) *p*-value *	4.35 ± 8.29	3.06 ± 6.51 0.61	3.86 ± 7.16 0.55	6.51 ± 10.6 0.01

* *p*-Values compared with preoperative values (*n* = 87 eyes) Mean ± SD. ITC: irido-trabecular contact, SD: standard deviation.

**Table 2:** Association between the presence of ITC and IOP increase after PKP.

	ITC (+)	ITC (-)	Total
**IOP increase (+)**	10	1	11
**IOP increase (-)**	28	48	76
Total	38	49	87

No. of eyes. Post-PKP IOP increase was defined as an increase in intraocular pressure above 21 mmHg. ITC: irido-trabecular contact, IOP: intraocular pressure, *p*-value = 0.0009 (Fisher’s exact test).

**Table 3:** Preoperative aqueous cytokine levels stratified by the presence of PAS after PKP.

	PAS (-) (*n* = 51)	PAS (+) (*n* = 36)	*p*-Value ^†^
Total protein	0.88 ± 0.72	0.92 ± 0.47	0.322
IL-1α	52.0 ± 106	88.1 ±166	0.438
IL-1β	2.59 ± 2.89	21.4 ± 64.1	0.214
IL-4	56.0 ± 75.0	79.8 ±118	0.084
IL-6	505 ± 1290	1290 ±3072	0.017
IL-8	26.6 ± 20.0	84.6 ± 150	0.008
IL-10	2.91 ± 9.43	7.85 ± 17.6	0.028
IL-12p70	7.59 ± 5.60	23.1 ± 36.2	0.014
IL-13	6.92 ± 2.25	9.01 ± 6.58	0.302
IL-17A	10.3 ± 10.0	23.1 ± 42.8	0.401
MIP-1α	18.2 ± 33.0	16.5 ± 11.8	0.078
MIP-1β	177 ± 244	191 ± 287	0.816
MCP-1	697 ± 317	904 ± 325	0.010
TNF-α	72.5 ± 65.4	69.0 ± 66.1	0.825
IFN-α	3.08 ± 1.89	2.83 ± 1.77	0.747
IFN-γ	99.6 ± 123	224 ± 489	0.097
E-Selectin	3890 ± 4990	4210 ± 3180	0.106
P-Selectin	5980 ± 5730	9700 ± 15,100	0.041
sICAM-1	3680 ± 3790	7160 ± 12,500	0.089
IP10	362 ± 966	394 ± 736	0.028
GM-CSF	7.11 ± 6.44	8.01 ± 8.00	0.871

Mean ± SD Protein; (mg/mL), Cytokines; (pg/mL). ^†^ Mann–Whitney U test, compared between eyes with and without PAS after DSAEK. PAS: peripheral anterior synechiae, IL: interleukin, MIP: macrophage inflammatory protein, MCP: monocyte chemotactic protein, TNF: tumor necrosis factor, GM-CSF: granulocyte-macrophage colony-stimulating factor, IFN: interferon, sICAM: soluble intracellular adhesion molecule, IP10: interferon gamma-induced protein 10.

**Table 4:** Correlations between preoperative cytokine levels and the PAS area after PKP.

	Preop PAS Area		PAS Area at 3 Months		PAS Area at 6 Months		PAS Area at 12 Months	
	r	*p*-Value ^*^	r	*p*-Value ^*^	r	*p*-Value ^*^	r	*p*-Value ^*^
IL-1α	0.006	0.964	0.073	0.591	0.049	0.742	0.116	0.414
IL-1β	0.165	0.291	0.140	0.394	0.017	0.927	0.056	0.750
IL-4	0.138	0.262	0.237	0.064	0.364	0.010	0.303	0.025
IL-6	0.385	0.001	0.312	0.014	0.387	0.007	0.396	0.003
IL-8	0.269	0.026	0.358	0.004	0.300	0.034	0.263	0.053
IL-10	0.341	0.010	0.173	0.207	0.235	0.144	0.251	0.100
IL-12p70	0.270	0.076	0.406	0.012	0.491	0.005	0.329	0.054
IL-13	0.218	0.239	0.163	0.428	0.052	0.815	0.168	0.403
IL-17A	0.190	0.229	0.286	0.087	0.191	0.320	0.171	0.342
MIP-1α	0.237	0.109	0.136	0.396	0.194	0.273	0.129	0.441
MIP-1β	0.105	0.388	0.078	0.542	0.001	0.993	0.033	0.809
MCP-1	0.138	0.247	0.151	0.231	0.378	0.005	0.330	0.012
TNF-α	0.004	0.982	0.028	0.864	−0.091	0.614	0.018	0.913
IFN-α	−0.069	0.681	−0.095	0.600	−0.097	0.622	−0.021	0.908
IFN-γ	0.211	0.134	0.271	0.069	0.395	0.015	0.378	0.015
E-Selectin	0.217	0.102	0.170	0.227	0.272	0.085	0.279	0.060
P-Selectin	0.071	0.553	0.100	0.428	0.282	0.041	0.171	0.198
sICAM-1	0.174	0.141	0.213	0.085	0.270	0.049	0.261	0.046
IP10	0.096	0.420	0.091	0.469	0.115	0.411	0.039	0.773
GM-CSF	−0.016	0.962	0.189	0.601	0.155	0.670	0.212	0.556

^*^ Spearman’s correlation analysis, Protein; (mg/mL), Cytokines; (pg/mL). IL: interleukin, MIP: macrophage inflammatory protein, MCP: monocyte chemotactic protein, TNF: tumor necrosis factor, GM-CSF: granulocyte-macrophage colony-stimulating factor, IFN: interferon, sICAM: soluble intracellular adhesion molecule, IP10: interferon gamma-induced protein 10.

## Data Availability

Data will be available if it is requested for proper reasons.

## Supplementary Materials

The following are available online at https://www.mdpi.com/article/10.3390/ijms222212268/s1, Table S1: Demographics of patients, Table S2: Multivariate analysis for clinical factors associated with progression of ITC area, Table S3: Correlations among the cytokine levels.
