# Supplementary material for: Elevated Cytokine Levels in Aqueous Humor Are Associated with Peripheral Anterior Synechiae after Penetrating Keratoplasty
Source: Int J Mol Sci. 2021 Nov 12;22(22):12268. doi: 10.3390/ijms222212268 (PMC8618311; doi:10.3390/ijms222212268)
Supplement: Supplementary file 1 [file ijms-22-12268-s001.zip › ijms-1420835-supplementary.pdf]

**Table S1.** **Demographics of patients**

| | |
|---|---|
| Eyes (n) | 87 |
| Sex, n (%) | |
| Male | 51(59%) |
| Female | 36 (41%) |
| Age (years) | 64.4±17.2 |
| range | (18-95) |
| Axial length (mm) | 25.0±2.62 |

Mean ± SD

SD: standard deviation, BSCVA: best spectacle-corrected visual acuity, logMAR: logarithm of minimal angle resolution, IOP: intraocular pressure, CCT: central corneal thickness, NA: not available

*Chi-squared test

**Table S2. Multivariate analysis for clinical factors associated with progression of ITC area**

| Δ ITC Area | 3 months | | 6 months | | 12 months | |
|---|---|---|---|---|---|---|
| | β | P value | β | P value | β | P value |
| **Preop total protein levels** | -0.789 | 0.526 | 0.912 | 0.528 | -0.108 | 0.729 |
| **Preop ITC (0 or 1) *** | **-3.91** | **0.017** | **-4.06** | **0.027** | **2.30** | **0.000** |
| **Axial length (mm)** | -0.145 | 0.618 | -0.528 | 0.137 | -0.023 | 0.747 |
| **Graft size (mm)** | 0.608 | 0.819 | 4.47 | 0.428 | -0.365 | 0.773 |
| **Age** | 0.013 | 0.760 | -0.005 | 0.926 | 0.008 | 0.454 |
| **Adjusted $R^2$** | 0.0187 | | 0.0471 | | 0.396 | |

VIF = 1.11~1.15

*Presence of preoperative ITC was dichotomized as categorical variables for multivariate regression analysis as follows: presence of preoperative ITC, absence = 0, presence = 1

ITC: irido-trabecular contact, VIF: variance inflation factor

ΔITC Area = (Postoperative ITC area) - (Preoperative ITC area)

## Table S3. Correlations among the cytokine levels.

| | MIP-1α | IL-1β | IL-4 | IP-10 | IL-6 | IL-8 | IL-10 | IL-12p70 | IL-13 | IL-17A | IFN-γ | GM-CSF | TNF-α | MIP-1β | IFN-α | MCP-1 | P-Sel | IL-1α | sICAM-1 | E-Sel |
|---|---|---|---|---|---|---|---|---|---|---|---|---|---|---|---|---|---|---|---|---|
| **MIP-1α** | | 0.272 | -0.126 | **0.373** | 0.147 | 0.258 | **0.463** | **0.452** | 0.307 | -0.216 | -0.332 | -0.285 | 0.268 | **0.450** | 0.132 | -0.021 | **0.374** | **0.513** | -0.258 | **0.371** |
| **IL-1β** | 0.114 | | -0.103 | 0.157 | 0.083 | 0.081 | 0.167 | 0.155 | -0.037 | 0.103 | -0.021 | 0.317 | 0.179 | 0.130 | 0.109 | -0.154 | **-0.090** | 0.295 | -0.139 | 0.013 |
| **IL-4** | 0.415 | 0.521 | | **0.256** | **0.252** | **0.356** | 0.172 | 0.132 | -0.172 | **0.393** | **0.470** | 0.280 | **-0.386** | 0.204 | **-0.236** | 0.475 | -0.127 | **0.310** | **0.520** | **0.341** |
| **IP-10** | **0.010** | 0.314 | **0.035** | | **0.308** | **0.355** | **0.485** | 0.293 | 0.134 | -0.014 | -0.003 | -0.168 | 0.147 | **0.438** | -0.047 | 0.136 | 0.147 | **0.447** | 0.161 | 0.049 |
| **IL-6** | 0.335 | 0.602 | **0.043** | **0.011** | | **0.463** | **0.493** | 0.250 | -0.010 | 0.152 | **0.352** | 0.143 | -0.026 | 0.111 | -0.146 | **0.295** | 0.059 | 0.242 | **0.406** | **0.271** |
| **IL-8** | 0.087 | 0.608 | **0.003** | **0.003** | **0.000** | | **0.449** | 0.140 | 0.011 | 0.240 | **0.328** | 0.335 | -0.052 | **0.359** | 0.086 | **0.292** | -0.020 | **0.364** | **0.287** | **0.282** |
| **IL-10** | **0.002** | 0.323 | 0.210 | **0.000** | **0.000** | **0.001** | | **0.305** | **0.504** | -0.064 | -0.142 | -0.238 | **0.364** | **0.341** | 0.107 | 0.041 | 0.025 | **0.565** | 0.106 | **0.288** |
| **IL-12p70** | **0.008** | 0.400 | 0.398 | 0.054 | 0.105 | 0.372 | **0.046** | | 0.158 | 0.192 | **0.437** | 0.143 | 0.240 | -0.189 | -0.034 | **0.350** | **0.329** | 0.068 | **0.317** | **0.438** |
| **IL-13** | 0.094 | 0.857 | 0.355 | 0.472 | 0.959 | 0.955 | **0.004** | 0.413 | | 0.055 | -0.063 | -0.029 | **0.557** | **0.439** | **0.542** | -0.210 | -0.192 | **0.361** | 0.000 | -0.141 |
| **IL-17A** | 0.214 | 0.562 | **0.011** | 0.929 | 0.335 | 0.126 | 0.705 | 0.285 | 0.775 | | **0.590** | 0.679 | 0.131 | -0.071 | -0.326 | 0.187 | 0.003 | -0.191 | **0.364** | 0.201 |
| **IFN-γ** | 0.052 | 0.901 | **0.001** | 0.983 | **0.011** | **0.018** | 0.340 | **0.003** | 0.741 | **0.000** | | 0.643 | -0.272 | **-0.307** | -0.040 | **0.560** | 0.247 | -0.368 | **0.725** | **0.550** |
| **GM-CSF** | 0.425 | 0.406 | 0.434 | 0.601 | 0.736 | 0.379 | 0.570 | 0.736 | 0.957 | 0.094 | 0.120 | | 0.500 | -0.242 | 0.323 | -0.368 | -0.116 | -0.295 | 0.133 | 0.395 |
| **TNF-α** | 0.095 | 0.288 | **0.011** | 0.329 | 0.868 | 0.737 | **0.019** | 0.186 | **0.002** | 0.427 | 0.090 | 0.171 | | **0.674** | **0.466** | **-0.560** | 0.008 | **0.534** | **-0.373** | -0.247 |
| **MIP-1β** | **0.002** | 0.408 | 0.100 | **0.000** | 0.379 | **0.003** | **0.012** | 0.226 | **0.014** | 0.657 | **0.028** | 0.448 | **0.000** | | **0.574** | -0.158 | -0.091 | **0.842** | -0.012 | -0.164 |
| **IFN-α** | 0.465 | 0.568 | 0.100 | 0.781 | 0.396 | 0.612 | 0.534 | 0.862 | **0.003** | 0.097 | 0.836 | 0.435 | **0.008** | **0.000** | | -0.145 | 0.028 | **0.395** | 0.123 | -0.161 |
| **MCP-1** | 0.893 | 0.323 | 0.160 | 0.254 | **0.015** | **0.015** | 0.763 | **0.020** | 0.259 | 0.235 | **0.000** | 0.239 | **0.000** | 0.193 | 0.393 | | **0.281** | -0.147 | **0.664** | **0.376** |
| **P-Sel** | **0.010** | 0.568 | 0.302 | 0.218 | 0.635 | 0.874 | 0.855 | **0.029** | 0.301 | 0.983 | 0.078 | 0.720 | 0.958 | 0.453 | 0.870 | **0.017** | | -0.089 | 0.120 | 0.219 |
| **IL-1α** | **0.000** | 0.061 | **0.018** | **0.000** | 0.070 | **0.005** | **0.000** | 0.703 | **0.046** | 0.231 | **0.016** | 0.353 | **0.000** | **0.000** | **0.016** | 0.255 | 0.491 | | -0.082 | -0.236 |
| **sICAM-1** | 0.080 | 0.374 | **0.000** | 0.176 | **0.001** | **0.017** | 0.435 | **0.036** | 0.998 | **0.018** | **0.000** | 0.680 | **0.011** | 0.925 | 0.462 | **0.000** | 0.316 | 0.524 | | **0.475** |
| **E-Sel** | **0.016** | 0.938 | **0.010** | 0.719 | **0.042** | **0.033** | **0.040** | **0.004** | 0.450 | 0.226 | **0.000** | 0.333 | 0.124 | 0.233 | 0.341 | **0.004** | 0.101 | 0.099 | **0.000** | |

Spearman's correlation analysis, IL: interleukin, MIP: macrophage inflammatory protein, MCP: monocyte chemotactic protein, TNF: tumor necrosis factor, GM-CSF: granulocyte-macrophage colony-stimulating factor, IFN: interferon, sICAM: soluble intracellular adhesion molecule, IP10: interferon gamma-induced protein 10

| | |
|---|---|
| | Correlation coefficient |
| P value | |
